# Healthy Eating in Low-Income Rural Louisiana Parishes: Formative Research for Future Social Marketing Campaigns

**DOI:** 10.3390/ijerph18094745

**Published:** 2021-04-29

**Authors:** Linda Fergus, Richie Roberts, Denise Holston

**Affiliations:** 1 School of Nutrition and Food Sciences, Louisiana State University AgCenter, Baton Rouge, LA 70803, USA; DHolston@Agcenter.lsu.edu; 2 Department of Agricultural and Extension Education and Evaluation, Louisiana State University, Baton Rouge, LA 70803, USA; Roberts3@lsu.edu

**Keywords:** rural population, low-income population, healthy eating, theory of planned behavior, social marketing

## Abstract

High rates of obesity and chronic disease exist in the southeastern United States (US). Knowledge about the attitudes, beliefs, and barriers of the rural low-income Louisiana population regarding healthy eating is limited. Focus Group discussions based on the Theory of Planned Behavior (TPB) were conducted in rural parishes (N = 3) with low-income residents of Louisiana (N = 29). Grounded Theory methods and cross-case analysis were used. The participants were primarily single Black females of age 18–30 years who earned a high school diploma, were employed, and had children. Beliefs included healthy eating was physically beneficial, yet financial impacts and the low palatability of healthy foods were barriers. Professional resources for nutrition education were limited which led to reliance on friends, family, and the internet. Friends and family were positive and negative influences on eating choices. Control beliefs included the high prices and low palatability of healthy foods, the wide availability of Energy Dense Nutrient Poor (EDNP) foods, and low motivation to sustain eating behavior changes. Formative research to optimize campaign distribution channels may improve accessibility to social marketing support and healthy eating resources. Persuasive messages that address control beliefs are needed in social marketing campaigns for rural low-income Louisiana environments.

## 1. Introduction

Consuming a healthy diet that adheres to the Dietary Guidelines for Americans (DGA) is associated with decreased mortality in residents of low-income areas of the southern United States (US) [1], even though higher obesity and chronic disease rates exist for the southeast US region [2,3,4,5]. Rural residents of the southeastern US exhibit sufficient knowledge of a healthy diet [6,7,8], yet knowledge frequently does not translate into adequate intake of fruit, vegetables, whole grain foods, and dairy products [9,10,11]. Environmental factors such as high prices of healthy foods [6,8,12,13,14,15] and shortages of quality produce [6,12,16] in rural stores reduce healthy food purchases. As a result, more rural residents are outshopping in search of variety, better quality, and lower prices of food [6,12,15,16]. One barrier to healthy eating is readily available Energy Dense Nutrient Poor (EDNP) foods [7,13,14,17]. The need for convenient meals [7,13,14], preferences of friends and family [6,7,14], and familiar taste predilections also influence food choices of low-income rural southeast US residents [6,8,14]. People living in rural and remote low-income environments outside of the US report high prices of healthy foods [18,19,20,21], low quality of produce [18,20], readily available highly palatable EDNP foods [18,19,20,21,22], and the importance of maintaining traditional ways to obtain food, such as hunting, fishing, or gardening [18,22]. 

The Supplemental Nutrition Assistance Program-Education (SNAP-Ed) provides direct nutrition education, comprehensive multi-level interventions, and community approaches (e.g., social marketing campaigns) to assist SNAP-eligible clientele to reach the DGA on a limited budget [23]. SNAP-Ed social marketing campaigns designed to promote healthy eating demonstrate improved attitudes, such as positive beliefs and readiness to eat fruit and vegetables, and healthy behaviors (i.e., increased intake of lower fat milk among SNAP recipients) [24,25,26]. According to Andreasen, “Social marketing is the adaptation of commercial marketing technologies to programs designed to influence the voluntary behavior of target audiences to improve their personal welfare and that of the society of which they are a part [27].” Beginning in 2014, nutrition educators at Louisiana Cooperative Extension Service developed and implemented two consecutive social marketing campaigns to promote healthy eating and improved PA throughout Louisiana. An evaluation of Louisiana’s 2017-2018 campaign recommended improvements to the campaign’s reach for the young adult population via social media, increased television exposure, and more diversity in print media and recipes [28,29,30]. It is important to promote healthy habits in young Louisiana residents because health behaviors are established in youth and continue throughout life [31]. In Louisiana, 18–19-year-olds often reside with their parent(s) if their high school completion is prolonged, which was the case for 20% of Louisiana high school students in 2018 [32]. Since adolescents are influenced by both their peers and parents, persuasive messages may need to address peer and parental norms and perceived behavioral control factors unique to adolescents [33,34].

Social marketing campaigns targeting low-income audiences have recently begun to use text messaging, internet, and social media to disseminate nutrition education, recipes, and supportive messages [24,25,35,36]. Access to social support through social marketing campaigns may motivate adults to act on intentions consistent with healthy eating behaviors [35,37]. Twenty-five percent of Louisiana households lack internet access, and 42% lack broadband connectivity which has deterred the provision of social marketing campaign materials, nutrition education, and social support via the internet [38]. Recently, the Louisiana legislature established a task force to improve broadband connectivity throughout Louisiana, especially in rural areas.

During the development of social marketing campaigns, researchers assess the target population’s beliefs and attitudes to optimally position the campaign messages with the intended audience [15]. Formative research is also used to identify targeted foods, behaviors, and ideal timing of persuasive messages and interventions [39,40,41]. Although research about healthy eating behaviors is available concerning rural communities in the southeast US [6,7,8,12,13,14,15,16], little is known about the barriers, attitudes, and beliefs of the rural, low-income population in Louisiana. To address this deficiency in knowledge, further investigation is needed to describe factors related to healthy eating behaviors. Using the Theory of Planned Behavior (TPB) [42], this research aimed to give details about the attitudes, subjective norms, perceived behavioral control beliefs, and intentions of the rural low-income Louisiana population regarding healthy eating to inform future social marketing campaigns in this state.

Fishbein and Ajzen introduced the Theory of Reasoned Action to demonstrate that behavior is based on volition and intention; thus, it postulates that behavioral and normative beliefs are key in the formation of a behavioral intention, which may result in a behavior change [43]. In 1985, Ajzen furthered this theory by adding perceived behavioral control to account for the resources and opportunities that the person believes they possess to change the behavior [44]. Known as the TPB, it suggests that human behavior is steered by the combination of (1) *behavioral beliefs* leading to attitudes about the behavior, (2) *normative beliefs* leading to subjective norms or social expectations to execute or not execute the behavior, and (3) *control beliefs* leading to perceived behavioral control or self-efficacy to perform the behavior. These factors coalesce to form an individual’s behavioral intention, which leads to the performance of the behavior if enough actual behavioral control exists [42]. The TPB is often used by social marketers to assess individual factors that influence planned behaviors (i.e., barriers) in order to develop persuasive messages and approaches that shape beliefs which may improve the performance of the desired behavior [45]. The TPB has been employed in the assessment of nutrition behaviors in rural Virginia [46] and the rural mid-Western US [47], while also being used as a framework for nutrition interventions in low-income populations [48,49,50]. The TPB is widely researched and shown to be an appropriate framework to assess and promote healthy eating behaviors of rural, low-income populations in the US.

## 2. Materials and Methods

A multiple case study approach in Louisiana parishes (N = 3 cases) with focus group discussions (FGD) was used [51]. Working from a constructivist epistemology with Grounded Theory methods, an inductive process to categorize and develop themes from the data was employed. Applying the more flexible Charmaz method of Grounded Theory encouraged the exploration of multiple realities of participants [52,53].

A purposive sample was obtained by identifying SNAP-Ed census tracts in rural parishes delineated by levels 4–9 of the Rural-Urban Continuum Codes (RUCC) [54]. SNAP-Ed nutrition educators arranged two FGD in North Louisiana (Winn Parish and Concordia Parish) and one FGD in South Louisiana (Washington Parish), and the nutrition educators recruited community residents who were at least 18 years of age, English-speaking, and willing to give consent to participate. Winn Parish, Concordia Parish, and Washington Parish have poverty levels of 18.7%, 25%, and 24.4%, respectively, and the Louisiana poverty level is 19.2% [55]. Additionally, these three parishes have obesity and diabetes prevalence rates that exceed the Louisiana rates for obesity (34.52%) and diabetes (11.1%) [56,57].

The TPB guided the development of 10 open-ended FGD questions to identify new information about attitudes, beliefs, barriers, and intentions of the rural, low-income audience regarding eating habits and making changes to nutrition behaviors [42,58]. These questions were peer-reviewed (Appendix A). Data about physical activity is not presented in this paper. The informed consent and opening FGD remarks included a discussion of all matters related to procedural ethics including the privacy of data. Participants signed the Informed Consent Forms and entered personal demographic data, including name, age, city of residence, parish of residence, number of children in household under 18 years old, gender, racial or ethnic group(s), SNAP benefit participation, occupation, and education level into Qualtrics XM Software (Qualtrics software, Version-March 2019, Provo, UT, USA, 2019) which was loaded onto 4 tablet devices (Apple iPad mini^®^). A digital recorder was used to record the FGD which lasted 75 min. After the FGD, field notes were written and digital pictures of the venue were taken. Before the analysis, the recorded data was transcribed into text and the accuracy of the recordings and the verbatim text were verified.

Initially, the data for the three parishes were coded (Dedoose 8.3.35, SocioCultural Research Consultants, LLC, Los Angeles, CA, USA, 2020) using in vivo, emotion, and values codes [59]. In vivo coding uses the participants’ own words for the codes. In this case, in vivo coding provided observations on the language of the participants. Then, emotion codes were applied to capture the feelings associated with the discussion. Last, the data were coded according to the constructs of the TPB and the values of the participants. After this, pattern coding, a form of axial coding, was used to group the initial codes into concepts and condense the categories [59]. Pattern coding was manually applied to arrange the codes into 10 categories based on the TPB including one category about behaviors, attitudes, and values related to grocery shopping. Fourteen themes about healthy eating emerged from the pattern coding and were categorized into four constructs of the TPB.

High standards of qualitative research were maintained by implementing Tracy’s criteria [60]. Developing FGD questions based on TPB and obtaining peer-review ensured a high level of study rigor. Three FGD in rural Louisiana were conducted and data was collected to a point of theoretical saturation which was evident when no new information emerged [61]. The data were triangulated through field notes and multiple FGD followed by the separate analysis of each FGD and cross-analysis to identify themes. The three initial FGD coding results from the in vivo, emotion, and TPB with values coding and 1 final coding with pattern coding were confirmed for all documents, including the transcript, field notes, and FGD notes to ensure the credibility of the results.

## 3. Results

Focus group discussions were conducted from March–April of 2019 in Winn Parish (N = 16), Concordia Parish (N = 7), and Washington Parish (N = 6). The participants were primarily single Black females of age 18–30 years who earned a high school diploma, were employed, and had children living in the home (Table 1). Most participants (86%) resided in SNAP-Ed eligible census tracts, and 39% of the participants reported receiving SNAP benefits. Most people (N = 26) reported that they had responsibilities for shopping and/or preparing food. Five of the six participants in Washington Parish were students preparing for the General Educational Development exam. Thirteen percent of the participants were residents of a halfway house which supported the development of life skills including grocery shopping and cooking. These two sub-groups exist routinely in the Louisiana SNAP-Ed population. The following sections describe the emerging themes related to attitudes, subjective norms, perceived behavioral control beliefs, and intentions about healthy eating of the low-income, rural population of Louisiana.

### 3.1. Attitudes

The participants defined the term “healthy eating” as consuming specific foods, such as fruit, vegetables, lean proteins, whole grains, nuts, and foods prepared with less fat or salt in designated portion sizes. Washington Parish participants voiced questions about what foods were healthy in addition to fruit and vegetables. Healthy eating was described as having negative financial impacts when the price was considered high or when perishable foods were not consumed. One participant said, “I think when I try to do healthy stuff, I am about to spend a lot of money and I am fixing to have to go back before the end of the week because if it doesn’t get eaten, it is going to go bad.” Healthy food was depicted as less palatable relative to EDNP foods. One participant said, “I like to eat good. Like good food, right? I eat a salad here and there.” However, healthy food was explained as having both short-term benefits (i.e., “not tired all the time,” “better shape,” “feel better”) and long-term benefits (i.e., “live longer” and “positive effect on children’s health”). Most participants reported satisfactory access to healthy foods stocked in local shopping venues. However, a few participants who resided in the most rural areas described traveling one hour every week to buy groceries which limited purchases of fresh produce.

### 3.2. Subjective Norms

Professional sources of nutrition information included local physicians and nutrition educators with SNAP-Ed. Participants said that the doctors, “always give you a sheet and a guide.” As professional resources for nutrition information were limited, some participants relied on internet sources, family members, and prior knowledge gained in school health classes. When discussing the internet, one participant asked, “How in the world do you know if it’s a good site?” Another stated, “Yeah, people can put anything on the internet.” Referring to prior health classes, another participant said, “The Food Guide Pyramid is what sticks in my mind. Your protein, your eggs, and what not.” One participant said his family was a good source of nutrition information and included a grandmother who had “a great big garden. That’s how I know about vegetables. I would have to move to a big farm to have all that.” An older brother was a “freak with being healthy.” One participant obtained nutrition information from her father who was a marathon runner and weightlifter.

In general, the participants conveyed that friends and family were positive, negative, and neutral influences on healthy eating. Regarding family, one participant said, “Mine would be happier (if I ate more fruits and vegetables) because my kids are worried about my health.” In Winn Parish, participants reported that the parents and grandparents limited the intake of sweets for the young children, while the adult children encouraged the grandparents to eat healthy. The preferences of family members or others at shared meals influenced the healthiness of the food served. One participant said a barrier to healthy eating was, “somebody or a family member telling you that they don’t want that (healthy food). So, you have to take another route and find something else to cook in place of that.” Some participants said that family members would refuse to eat fruits and vegetables because they did not like them. Other participants conveyed that if they improved their eating behaviors, some members of the family would not notice. The participants in Washington Parish described that friends were a negative influence on healthy eating. When asked what their friends would say if they chose to eat more fruits and vegetables, the participants mimicked their friends’ response, “What is wrong with you?” or “He (boyfriend) would be like ‘Who you trying to get skinny for?’” One participant stated, “A lot of people don’t eat healthy. They like to go to Taco Bell, love to go to Burger King, go eat at that fast-food restaurant and spend their money, McDonalds.”

### 3.3. Perceived Behavioral Control

The price of healthy food was the most frequently mentioned barrier in Winn Parish and Concordia Parish. Additionally, the high palatability and availability of EDNP foods, particularly when leaving the workplace hungry, was a barrier to healthy food consumption. One participant said, “Yeah, you want to eat right then and there.” Washington Parish participants articulated impulsive urges to purchase unhealthy foods in the grocery store. One participant said, “I love chips, so the salad is going to have to wait.” In many instances, participants reported that healthy food does not meet taste expectations. One participant said, “Greens are vegetables, but the meat that we season our greens with is not the best kind of meat for us. But, if we could find a way to make it still taste as good as we want it to taste, I think everybody would eat more vegetables that way.” Participants also talked about the lack of motivation to sustain healthy behaviors, saying, “I could eat healthier. But I still have to have my chocolate,” and “I can eat healthy, but I don’t know how long I would keep it up.”

### 3.4. Intentions

Participants described mixed intentions related to healthy eating. When asked about planning to eat healthy foods, one participant stated, “Well, I have a plan. I just have to follow through with it. I made plans my whole life, but they don’t always work out.” One participant said, “Maybe you could have one cheat day out of the week.” Participants voiced the importance of planning to purchase healthy foods that would also satisfy taste preferences. Another participant said, “You need to do something like a challenge. You know like on Facebook (for support).” Some participants thought it was important to prepare several meals ahead of time, learn healthy food preparation methods, eat breakfast, keep healthy foods and snacks available, make gradual changes, and buy new cooking equipment (e.g., an air fryer or a dehydrator). Others thought that it was important to stop engaging in certain practices, such as frying foods, drinking sugar-sweetened beverages, and buying or preparing unhealthy foods.

### 3.5. Cross-Case Analysis

A cross-case analysis of themes classified by attitudes, subjective norms, perceived behavioral control beliefs, and intentions aided in the development of final themes through comparison and contrast of findings in each parish. The themes were consistent across parishes. However, one group of participants did not mention the long-term benefits of healthy eating or high prices of healthy foods as a barrier (Table 2).

## 4. Discussion

The findings of this study provide insight into the attitudes, subjective norms, perceived behavioral control beliefs, and intentions of rural, low-income Louisiana residents regarding healthy eating behaviors. One pervasive attitude was that healthy eating has negative financial impacts primarily due to the price of substituting healthier foods for unhealthy foods. Price has previously been identified as a major barrier to healthy eating in the US [6,8,12,13,14] and outside of the US [18,19,20,21,22]. In this study, participants shopped at discount stores or outside of their local community (i.e., outshopping) to obtain better prices and quality foods. Healthy foods were perceived as less palatable relative to EDNP foods, and serving healthy foods sometimes resulted in uneaten foods and subsequent financial losses due to waste. Concordia Parish and Winn Parish participants described healthy eating consistently with the DGA including the type of food, preparation methods, and food portion controls similarly to previous findings in US rural populations [6,7,8]. Every FGD identified the short-term advantages of healthy eating as physiologically beneficial. Participants in Concordia Parish and Winn Parish also identified long-term benefits of healthy eating as noted in other studies [8,17,62]. These emergent findings are consistent with other studies about healthy eating in the rural low-income populations of Louisiana [12] and Mississippi [6,8,17]. Higher rates of chronic health conditions exist in the deep south compared to other regions of the US [4], and unique attitudes, beliefs, and barriers to healthy eating may be contributing factors.

Two themes were identified related to the influences of subjective norms on eating. First, limited availability of health care professionals and nutrition educators led to reliance on other information sources, such as the internet (if available), friends, and family. Second, family and friends were described as positive, negative, and neutral influences on healthy eating. Participants in Winn Parish discussed a mutually beneficial interaction between the adults and the children, where the parents and grandparents limited intake of sweets for the younger children, and the adult children encouraged healthy eating in the older adults. However, family members were a negative influence when they refused to eat healthy foods. In Washington Parish, friends were a negative influence on healthy eating during socialization with peers while eating fast-food. Additionally, participants described teasing from peers when they attempted to eat healthfully.

Perceived behavioral control beliefs included the availability of low cost, EDNP fast foods, high prices of healthy foods, lower palatability of healthy foods compared to EDNP foods, and low motivation to sustain healthy eating behaviors. The high price of healthy foods [6,8,12,13,14] and the availability of EDNP foods [7,13,14,17] have been previously identified as barriers to improving eating behaviors in the southeast US. Participants expressed interest in eating highly palatable EDNP foods which they termed as “bad” foods. Consumption of EDNP foods frequently occurred in conjunction with leaving work for the day and was similar to previous findings in the rural, low-income population where the need for convenience and instant gratification were cited [7,13,14,17]. Food purchases and meal preparation were influenced by the food preparer’s energy level, hunger intensity, the anticipated length of time to prepare a home-cooked meal, family schedules, and/or emotional drivers for eating. Eating fast-food was an easy choice when compared to the alternative of preparing a healthy meal at home which was not perceived to be convenient or preferable in taste for everyone.

The intentions to eat healthy foods ranged from highly motivated to begin new behaviors to not motivated or overwhelmed. Winn Parish participants wanted to learn to prepare meals ahead or begin “meal prepping” for several days in advance. Participants who intended to improve their eating suggested facilitators such as social support and planning ahead would enhance the likelihood of engaging in new healthy behaviors.

### 4.1. Study Limitations

Limitations of this research include using the purposive sampling method which may have led to selection bias and nonrepresentation of the population; however, 86% of participants resided in rural SNAP-Ed eligible census tracts. Participants were primarily single women between 18–30 years old. Due to the demographics of the sample, this study may not reflect the needs of males, residents outside of rural areas in the southeast US, and populations under different socioeconomic conditions.

### 4.2. Implications for Research and Practice

The study participants described control beliefs about eating healthy foods including time constraints, restricted budgets, taste expectations, and limited support to increase and sustain motivation for new behaviors. Due to limited availability of professional nutrition education resources in rural parishes, participants relied on friends, family, and the internet (if available) for healthy eating information. Two themes, social support and planning, enhanced the likelihood of acting on healthy eating intentions. Prior recommendations from a recent social marketing campaign evaluation in Louisiana included improving the campaign’s reach in the young adult population via social media [28]. Currently, plans for broadband connectivity improvements in rural Louisiana provide the opportunity to increase support for healthy eating behaviors and nutrition education (including meal planning) in future social marketing campaigns. Formative research is needed to identify and prioritize new social marketing distribution channels (i.e., text messaging, internet, and social media) in combination with current distribution channels (e.g., print and broadcast media) and to mutually determine the targeted foods, beverages, and behaviors to address. Since participants reported the barriers of time constraints, price, and taste concerns of healthy foods, it will be important to identify the specific barriers to each targeted food and address the obstacles in relevant persuasive messages [26]. Similar to residents of remote environments outside of the US [18], some Louisiana residents report hunting and fishing to reduce food expenses [12]. Other solutions using local natural resources may be identified to reduce barriers to healthy eating.

Two targets for persuasive messages and community education are the “good food–bad foods” and the “cheat day” approaches to dieting. These tactics may lead to increased hunger and feelings of deprivation due to the omission of preferred foods and may trigger subsequent over-eating. Reinforcing the Academy of Nutrition and Dietetics position on the total diet approach, which includes maintaining a healthy diet pattern as a lifestyle change, may improve long-term eating habits [63]. Education about consuming meals that are inclusive of all foods, eating in moderation, trying new foods, and following healthy portion sizes is continually needed. Additional supportive interventions (e.g., social media challenges, group cooking and educational opportunities, and healthy recipes that meet taste expectations) may enhance future campaign participation by Louisiana rural populations.

Another target for persuasive messages is the frequent purchase of fast foods at dinner meals. Bacon found that lower-income and single parents preferred a health frame more often than a family togetherness frame for acknowledging the importance of family meals [37]. Persuasive messages which emphasize the long-term benefits of home-cooked meals, (i.e., health of the family and lower cost of meals) may be beneficial. More nutrition education and support for healthy shopping (e.g., grocery shopping phone applications) and food preparation (e.g., how to plan, shop, prepare, and safely store multiple meals) geared to single parent families may increase healthy eating in Louisiana. Consumption of EDNP foods at meals is not unique to the US. Residents of rural and remote environments in Canada, Uganda, Australia, and the Pacific Islands countries reported that EDNP foods are more available due to increased trade. Consequently, EDNP foods are considered a barrier to the consumption of healthy traditional foods in their respective cultures [18,19,20,21,22]. Healthy eating promotions in these remote locations encourage the intake of traditional healthy foods shared with family and friends for the prevention of obesity and chronic disease [18,21].

Food choices may be influenced by both peer and parental beliefs while adolescents complete their high school education. This was seen in Washington Parish where most of the participants were 18–19 years of age and half reported their mothers prepared some meals for them. Participants in Washington Parish asked the most questions about which foods were nutritious. These adolescents described more impulsive behaviors and less behavioral control while shopping than participants in other FGD. Since behavioral control beliefs have a strong correlation with intention [34], persuasive messages to target impulsivity in stores may be beneficial. The adolescent participants believed that their peers were eating in fast food restaurants frequently; however, adolescents may misperceive the behavior of their peers. Social marketing messages designed to simultaneously clarify or change the social norms of peers and support healthy parental norms may be effective [33]. Additional formative research and segmentation of future social marketing campaigns may be necessary to provide tailored nutrition education and to achieve optimal message resonance with older adolescents.

## 5. Conclusions

In conclusion, this research demonstrated that the TPB was an effective theory from which to elicit healthy eating attitudes, beliefs, and barriers of low-income rural residents, including SNAP-Ed participants in Louisiana. Eating attitudes, beliefs, and barriers of the target audience included budgetary concerns, highly available and palatable EDNP foods, the low palatability of healthy foods, and decreased motivation to sustain healthy eating behaviors. Formative research to clarify optimal distribution channels may improve the reach of future social marketing campaigns, and larger reach may increase support for changes in eating behaviors. Additionally, formative research to mutually establish healthy food and beverage targets for intervention and to investigate the control beliefs associated with the targets is essential for the development of persuasive messages. Future social marketing campaigns may benefit from additional research and segmentation of the population to tailor social marketing messages, nutrition education, and interventions to older adolescents. Research findings from this study will be used to inform the development of future SNAP-Ed social marketing campaigns to promote healthy eating behaviors by rural low-income residents in Louisiana.

## Figures and Tables

**Table 1:** Demographic information for FGD participants in three rural low-income Louisiana parishes, N = 28 ^a^.

Variable.	N	%
**Gender**		
Female	24	86
Male	4	14
**Ethnicity/Race**		
Black	15	54
White	11	39
American Indian or Alaskan Native	1	3
Other	1	3
**Education**		
Less than high school	10	36
High school or GED ^b^	14	50
Some college	2	7
College degree	2	7
**Marital Status**		
Married	4	14
Unmarried couple	4	14
Single	16	57
Divorced	3	11
Separated	1	3
**Age (years)**		
18-30	16	57
31-40	2	7
41-50	3	11
51-60	4	14
61-67	3	11
**Do you receive SNAP benefits?**		
Yes	11	39
No	17	61
**Number of children < age 18 years old who live in house**		
0	10	36
1	7	25
2	5	18
3	2	7
4	1	3
5	1	3
6 or more	2	7

^a^ One participant did not complete the demographic survey. ^b^ General Education Developmental exam.

**Table 2:** Cross-case analysis of qualitative themes related to healthy eating in three rural, low-income Louisiana parishes.

Themes	Washington	Concordia	Winn
**ATTITUDES about healthy eating**			
Negative financial impacts	X ^a^	X	X
Defined as control of foods and portions	X	X	X
Less palatable relative to EDNP ^b^ foods	X	X	X
Short-term physical benefits	X	X	X
Long-term physical benefits		X	X
			
**SUBJECTIVE NORMS for nutrition information**			
Limited professional resources led to reliance on other sources of nutrition information.	X	X	X
			
**SUBJECTIVE NORMS of family/friends**			
Friends and family may be positive, negative, and/or neutral influences on healthy eating.	X	X	X
			
**PERCEIVED BEHAVIORAL CONTROL-barriers to buying and eating healthy foods**			
High price of healthy foods		X	X
High palatability of EDNP foods	X	X	X
High availability of EDNP foods	X	X	X
Low palatability of healthy foods	X	X	X
Low motivation to sustain behavior change	X	X	X
**INTENTIONS**			
Social support enhances eating intentions.	X	X	X
Planning enhances healthy eating intentions.	X	X	X

^a^ “X” delineates the presence of the theme ^b^ Energy Dense Nutrient Poor.

## Data Availability

The data used/analyzed for this study are available from the corresponding author upon request.

## Appendix A

**Table A1:** Questions for focus group discussions in three low-income, rural Louisianaparishes.

TPB Construct	Question
Attitude	What do you think of when you hear the words “healthy eating”?
Attitude/SN	Who do you ask or “look to” for guidance about what to eat? Probe.
Attitude	If you chose to eat healthy, how would it affect you and your life?
Subjective norms	If you chose to eat more fruits and vegetables what would your family say? What would your friends say?
Perceived Behavioral Control	If you wanted to buy and prepare healthy foods, what barriers or obstacles may prevent you from being successful? Probe.
Perceived Behavioral Control	If you wanted to eat healthy, would you be able to? Why or why not?If you wanted to be physically active, would you be able to? Why or why not?
Attitude/SN	What would it be like or feel like if you were to change your eating habits or your physical activity habits? What would your family and friends say?
Intention	Do you have a plan to eat more healthfully? Why or why not? If so, how would you make it happen?
Intention	Do you have a plan to get more physical activity? Why or why not? If so, how would you make it happen?
